# The Effects of Four Weeks of Chiropractic Spinal Adjustments on Blood Biomarkers in Adults with Chronic Stroke: Secondary Outcomes of a Randomized Controlled Trial

**DOI:** 10.3390/jcm11247493

**Published:** 2022-12-17

**Authors:** Heidi Haavik, Imran Khan Niazi, Imran Amjad, Nitika Kumari, Usman Rashid, Jens Duehr, Muhammad Samran Navid, Robert J. Trager, Muhammad Shafique, Kelly Holt

**Affiliations:** 1 Centre for Chiropractic Research, New Zealand College of Chiropractic, Auckland 1060, New Zealand; 2 Faculty of Health & Environmental Sciences, Health & Rehabilitation Research Institute, AUT University, Auckland 1010, New Zealand; 3 Department of Health Science and Technology, Aalborg University, 9220 Aalborg, Denmark; 4 Faculty of Rehabilitation and Allied Health Sciences and Department of Biomedical Engineering, Riphah International University, Islamabad 46000, Pakistan; 5 Connor Whole Health, University Hospitals Cleveland Medical Center, Cleveland, OH 44106, USA

**Keywords:** chiropractic, physical therapy, brain-derived neurotrophic factors, glial cell-derived neurotrophic factors, insulin-like growth factor-II, stroke, spinal manipulation

## Abstract

Certain blood biomarkers are associated with neural protection and neural plasticity in healthy people and individuals with prior brain injury. To date, no studies have evaluated the effects chiropractic care on serum brain-derived neurotrophic factor (BDNF), insulin-like growth factor-II (IGF-II) and glial cell-derived neurotrophic factor (GDNF) in people with stroke. This manuscript reports pre-specified, exploratory, secondary outcomes from a previously completed parallel group randomized controlled trial. We evaluated differences between four weeks of chiropractic spinal adjustments combined with the usual physical therapy (chiro + PT) and sham chiropractic with physical therapy (sham + PT) on resting serum BDNF, IGF-II and GDNF in 63 adults with chronic stroke. Blood samples were assessed at baseline, four weeks (post-intervention), and eight weeks (follow-up). Data were analyzed using a linear multivariate mixed effects model. Within both groups there was a significant decrease in the mean log-concentration of BDNF and IGF-II at each follow-up, and significant increase log-concentration of GDNF at eight-weeks’ follow-up. However, no significant between-group differences in any of the blood biomarkers at each time-point were found. Further research is required to explore which factors influence changes in serum BDNF, IGF-II and GDNF following chiropractic spinal adjustments and physical therapy.

## 1. Introduction

Stroke is one of the leading causes of long-term disability and has significant physical, emotional, social and financial consequences on survivors and their families [1,2,3]. Spontaneous and rehabilitation-driven recovery is critical for improvement in post-stroke function [4,5,6]. The underlying mechanisms of recovery include cascades of cellular and molecular processes that induce neurogenesis, angiogenesis and neuroplasticity [7]. There is growing interest in understanding the changes in biomarkers accompanying stroke recovery, which may help personalize and improve stroke treatments [8,9].

Blood biomarkers are indicators found in blood that reflect underlying molecular or cellular events of behavioral state, disease state, or recovery, and may be used to examine treatment mechanisms and response to therapy [8]. Biomarkers can be measured in the central nervous system (CNS) or peripherally (via serum or plasma). Biomarkers, such as brain-derived neurotrophic factor (BDNF), glial cell-derived neurotrophic factor (GDNF) and insulin-like growth factor-II (IGF-II), are proteins associated with neural protection and neural plasticity in both healthy and injured brains [10,11]. 

The BDNF protein belongs to the neurotrophin family of growth factors [12,13,14,15] and is active in the hippocampus and areas of the brain responsible for learning, memory and thinking [16]. Insulin-like growth factor-II (IGF-II) is a protein-based hormone that shares structural similarities to insulin [17]. It is expressed by neurons as well as myocytes (i.e., as a myokine), is secreted by the liver, and can cross the blood brain barrier (BBB) to influence metabolic and endocrine function in the brain [18]. Along with the IGF system that includes IGF-I, receptors (IGF-I, IGF-II, and the insulin receptor) and IGF binding proteins, IGF-II plays a role in growth, metabolism and nervous system development and function [19,20,21]. In addition, IGF-I and IGF-II play a role in the repair of muscles in response to injury or exercise through their expression in skeletal muscle cells [22,23]. Specifically, IGF-II contributes to the maintenance of neurogenesis in the supraventricular zone and the hippocampus [24,25]. Another neurotrophin, GDNF, is expressed in the neurons of a healthy adult brain, and can also be expressed in the injured or diseased brain [26]. Expression of GDNF can promote axonal survival and growth, but prolonged overexpression or ectopic expression of GDNF can lead to abnormal neuronal sprouting [26]. GDNF supports other neurons like spinal motoneurons [23] and brain noradrenergic neurons [24], and regulates the survival, migration and differentiation of several peripheral neurons [25,26].

Motor rehabilitation is critical to stroke recovery [27,28,29,30,31,32]. Accumulated evidence has shown that motor rehabilitation programs consisting of repetitive task-specific training, such as constraint-induced movement therapy function (CIMT), robotic training, and body weight-supported treadmill training, promote neural plasticity for post-stroke recovery [27,28,29,30,31,32]. Currently, evidence suggests that physical activity can modulate central levels of GDNF [33,34,35] and both central and peripheral levels of BDNF and IGF-II [36,37,38]. Studies using animal stroke models have shown that multiple sessions of aerobic exercise can increase central BDNF concentrations, while BDNF responses following functional exercises, such as reaching and CIMT, are inconsistent [37]. In humans, peripheral BDNF levels have mainly been evaluated after an aerobic, strength, or endurance exercise program [39,40]. A meta-analysis of 11 studies including 303 individuals with neurological conditions found low-quality evidence supporting increased levels of BDNF following a program of aerobic exercise, while the results for a single bout of aerobic exercise were mixed [41]. Only one fair-quality study has evaluated the effect of multiple sessions of aerobic exercise plus physical therapy care in post-stroke individuals and reported increased serum BDNF levels [42]. However, there are no studies that have evaluated the effects of a single or multi-session exercise on serum IGF-II or GDNF levels in people with stroke. 

Another intervention that may have the potential to enhance recovery after stroke is chiropractic care. Chiropractic care is a holistic health approach that focuses on correcting central segmental motor control (CSMC) problems in the spine, often referred to by chiropractors as vertebral subluxations, using spinal adjustments (i.e., manipulations) [43,44,45]. Stroke survivors commonly seek chiropractic care, potentially to treat painful musculoskeletal disorders such as myalgia that arise following stroke [46,47]. A study using national survey data (2017) from the United States reported that 9.2% of respondents with a stroke indicated they had seen a chiropractor in the previous 12 months [47]. Previously, case reports provided limited evidence to suggest that chiropractic spinal adjustments could potentially trigger a stroke. However, several large epidemiologic studies have instead found that there is no increase in risk of stroke following chiropractic spinal adjustments relative to primary care physician visits [48,49,50,51]. Chiropractic spinal adjustments therefore appear to be safe in the post-stroke population [47], provided there are no absolute contraindications to this therapy, such as cervical arterial dissection [52].

There is a growing body of evidence indicating that chiropractic spinal adjustments significantly influence the function of the CNS [44,53,54]. A single session of chiropractic spinal adjustment has been shown to modulate somatosensory processing, sensorimotor integration and motor control [44,53,55,56,57,58,59,60,61,62]. Sensorimotor integration is the ability of the CNS to integrate and transform sensory inputs from multiple sources within the body to task-specific motor actions [63]. Effective sensorimotor integration and accurate internal awareness of the position of limbs and body in space are important for learning new motor skills and recovering from CNS lesions [64,65,66,67,68]. 

Recently, chiropractic care has been shown to have a positive impact on motor function in chronic stroke patients [69,70]. In one study, a single session of chiropractic spinal adjustment significantly increased plantar flexor muscle strength in 12 stroke survivors [69]. In a follow-up study, when four weeks of chiropractic spinal adjustment was combined with physical therapy, it resulted in a greater improvement in motor function compared to physical therapy combined with sham chiropractic care [70]. Further research is now needed to investigate the mechanisms underlying these findings. 

While research on this topic is limited, previous studies have reported that the mechanical stimulation of a chiropractic spinal adjustment may produce a neuro-immunomodulatory response and affect several biomarkers [71]. One study found that chiropractic spinal adjustment produced significant alterations in interferon-gamma, interleukin (IL)-5 and IL6 [71]. Another study found that this therapy reduced urine levels of tumor necrosis factor alpha [72]. An animal-based study reported increases in mechano growth factor, a variant of IGF-1, after chiropractic spinal adjustment [73]. Importantly, previous researchers have suggested that chiropractic spinal adjustment may stimulate the release of several neurotrophins, including BDNF; however, this has not been confirmed to date [74]. Accordingly, we sought to explore the effect of chiropractic spinal adjustment on biomarkers relevant to stroke recovery in individuals with chronic stroke.

It is currently not known if chiropractic spinal adjustments affect blood biomarkers associated with neural protection and neural plasticity in people with stroke. When investigating this research question it should be acknowledged that current interventions, such as physical therapy, are known to be beneficial to the recovery of stroke survivors [75,76]. These accepted interventions should not be withheld when investigating the impact of a relatively novel intervention. Therefore, this study aimed to investigate the effects of four weeks of chiropractic spinal adjustments combined with the usual physical therapy, compared to physical therapy alone on BDNF, IGF-II and GDNF levels in people with chronic stroke.

The current study represents unpublished secondary outcomes from a previously published randomized controlled trial (RCT) which examined the primary clinical outcome of motor function in the same patient population, with stroke receiving an identical intervention as described in the present manuscript [70]. Although the two manuscripts are related, it was not feasible to report the current secondary outcomes in the original RCT manuscript as there were several biomarkers studied which had a distinct collection methodology, statistical analysis plan and analysis. Readers should interpret the present manuscript with the awareness that the related RCT findings, as reported previously [70], identified that four weeks of combined chiropractic spinal adjustment and physiotherapy resulted in statistically significant, and likely clinically relevant, improvements in motor function, compared to a control group.

## 2. Materials and Methods

### 2.1. Design and Setting

This manuscript describes unpublished, pre-planned, secondary outcomes of exploratory biomarker analysis from a previously conducted parallel group RCT, the “Chiropractic Care Plus Physiotherapy Compared to Physiotherapy Alone in Chronic Stroke Patients Trial” (clinical trial registry: NCT03849794). The main RCT investigated the effects of four weeks of chiropractic spinal adjustments combined with the usual physical therapy on motor function in people with stroke [70]. Data were collected at the Rehabilitation Center of Railway General Hospital, Rawalpindi, Pakistan from January to June 2019. The Riphah International University Research Ethical Review Committee approved the study (Riphah/RCRS/REC/000458). All procedures performed in this study were in accordance with the 1964 Helsinki Declaration and its later amendments or comparable ethical standards.

### 2.2. Study Participants 

Participants were recruited by telephone from the Railway General Hospital database. Potential participants were required to have suffered from a stroke at least 12 weeks prior to their participation in the trial and have previously completed a rehabilitation program at the hospital. To be eligible, volunteers had to have ongoing significant motor impairment, indicated by a score of 80 or less on the combined upper and lower limb Fugl-Meyer Assessment (FMA) of motor function [77]. Participants were ineligible if they showed no evidence of spinal dysfunction (i.e., no presence of vertebral subluxation indicators identified by a chiropractor), had absolute contraindications to spinal adjustments (history of spinal fracture, atlantoaxial instability, cervical arterial dissection, spinal infection, spinal tumor, or cauda equina syndrome) or previously had an adverse event in response to chiropractic adjustment(s). Written consent was obtained from all volunteers before participation in the study.

### 2.3. Interventions

The study interventions were either four weeks of chiropractic adjustments plus physical therapy (chiro + PT), or four weeks of sham chiropractic adjustments plus physical therapy (sham + PT). As this was an exploratory study, a standalone chiropractic intervention was not considered as it would have meant withholding an intervention known to be effective in order to evaluate a novel intervention [76]. 

#### 2.3.1. Chiropractic Intervention 

The chiro + PT group were assessed for CSMC problems by New Zealand registered chiropractors approximately three times per week for four weeks and adjusted when necessary. The clinical indicators for CSMC problems that were used in this study are routinely used by chiropractors when analyzing the spine and included tenderness to palpation, restricted intersegmental motion, asymmetric muscle tension and altered joint-play [78]. These clinical indicators have previously been shown to be reliable when identifying CSMC problems when used as part of a multidimensional battery of tests [78,79]. Chiropractic adjustments included high-velocity, low-amplitude thrusts or instrument assisted thrusts to any region of the spine or pelvic joints [80]. The choice of spinal level(s) to adjust was left to the discretion of the chiropractor and generally involved adjustments to multiple levels on each visit. Chiropractic visits lasted approximately 15 min and no other interventions were provided by the chiropractor. 

#### 2.3.2. Sham Chiropractic Intervention

Due to the manual nature of the intervention, blinding of participants in trials receiving physical interventions is usually challenging [81,82]. One advantage of performing this study in Pakistan was that chiropractic is relatively unknown [83]. A recent survey found that more than 67% of university students studying pharmacy in Lahore, Pakistan, were unaware that chiropractic care was related to spinal manipulation and that it is often used as a low back pain intervention [83]. This lack of knowledge about chiropractic provided a unique opportunity to study chiropractic’s effects with the enhanced potential of successful participant blinding. To reduce the impact of contextual effects on study outcomes, the control group received a sham chiropractic intervention. 

Participants in the sham + PT group also saw a chiropractor approximately three times per week for four weeks. The chiropractor performed a similar assessment as the chiro + PT group, however, no thrusts to the spine were applied. Instead, the participant was positioned as if a thrust was going to be provided, but no thrust was given. Alternatively, an adjusting instrument was set to the minimum setting and placed on the chiropractor’s hand or arm, lateral to the spine, and a clicking sound was produced with the instrument. To assess how effective participant blinding was, participants in both groups were asked to indicate whether they perceived they had received active chiropractic care after the four-week intervention period was complete. 

#### 2.3.3. Physical Therapy Intervention 

Approximately 40 min of intensive physical therapy intervention was delivered three times per week to both the groups during the four-week intervention period. The physical therapy program consisted of muscle stretching and strengthening, sitting and standing balance training, sit-to-stand practice, transfer practices relative to patient needs, walking, stair climbing, upper limb functional training (reach, grasp and hand to mouth activities), muscle tone inhibition techniques, postural stability control, sensory techniques and functional daily activities. Occasionally, depending on the participants requirements, hot packs and transcutaneous electrical nerve stimulation were used to reduce pain or promote muscle relaxation [84]. Participants were also encouraged to continue performing exercises at home where appropriate. The physical therapists providing care in the study were experienced and qualified in treating neurological disorders. The physical therapy intervention did not include any spinal manipulation or mobilization. 

### 2.4. Outcome Measures

Serum levels of BDNF, IGF-II and GDNF were used as outcome measures. Levels of serum BDNF, IGF-II and GDNF were assessed prior to the intervention, at four weeks of the intervention and at eight weeks as a follow-up assessment. A five-milliliter venous blood sample was collected in an anticoagulant free tube (EDTA K3). Samples were kept for an hour at 4 °C before the serum was isolated. Serum was then stored at −80 °C for batch assessment. Serum levels of BDNF, IGF-II and GDNF were measured by a sandwich, two-site enzyme linked immunoassay (ELISA) using the BDNF, IGF-II and GDNF Elabscience^®^ Immunassay System reagents. Optical density (OD) was determined by using a micro-plate reader set to 450 nanometers. Four-parameter logistic curves were plotted between standard concentration and OD values. These procedures were performed at the Riphah Institute of Pharmacology Sciences, Riphah International University, Islamabad, Pakistan.

### 2.5. Randomization and Blinding

Following assessment for eligibility, an online minimization tool (QMinim, Telethon Kids Institute, Perth, Australia) was used to randomly assign participants to the chiro + PT or sham + PT group [85]. Fugl-Meyer Assessment (FMA) score, gender and age at baseline were entered as prognostic factors for minimization. All participants, outcome assessors and physical therapists providing the physical therapy intervention were blinded to group allocation. The technician who ran the ELISA procedure, the data analysts and the statistician who analyzed the data were also blinded to group allocation. This was accomplished by allocating a code to all the recorded data prior to sending for analysis. The chiropractic providers could not be blinded to group allocation. 

### 2.6. Statistical Analysis

The null hypothesis for this study was that there would be no difference between groups in any of the blood biomarker concentrations at either of the two post-intervention time-points. Blood biomarker concentration data was collated in Microsoft Excel (Microsoft Corp. Redmond, WA, USA) and exported to R statistical computing environment (version 4.02) for analysis [86]. A detailed report of the statistical analysis is available as a Supplementary File.

Normality of blood biomarker concentrations was evaluated using QQ-plots. The concentration for all biomarkers—BDNF, GDNF and IGF-II—was not normally distributed. To mitigate the non-normality, natural log transformations were applied, and the remaining analysis was conducted and reported using log-concentrations. The blood biomarker concentration along with independent variables including biomarker type, participants, group and time comprised a hierarchical longitudinal data structure having within-participant correlations and loss-to-follow-up. 

To match the needs of the data structure, a linear multivariate mixed effects model was constructed [87]. This model included log-concentration from the three biomarkers as the dependent variables. It had Outcome (BDNF, GDNF, IGF-II), Time (Baseline, four weeks, and eight weeks), Group (chiro + PT and sham + PT), and their two-way and three-way interactions as fixed effect independent categorical variables. Baseline was added as a time point rather than as a covariate because the concentration data were found to lack a linear relationship between baseline and post-intervention values. To cater to within-participant correlations, a random effects variance-covariance structure was also added. This random-effects structure estimated random intercepts for each participant separately for each outcome and allowed for between-outcome correlations. Positive definiteness was the only constraint on this structure which allowed correlations to take any value. From this model, a multivariate analysis of variance table was generated. Between-group mean differences and within group means were also estimated along with their standard errors and 95% confidence intervals. Between-group differences were computed at the four-week and eight-week time points while subtracting the baseline mean values to adjust for baseline differences. The statistical significance level was set at 0.05.

## 3. Results

**Figure 1:**
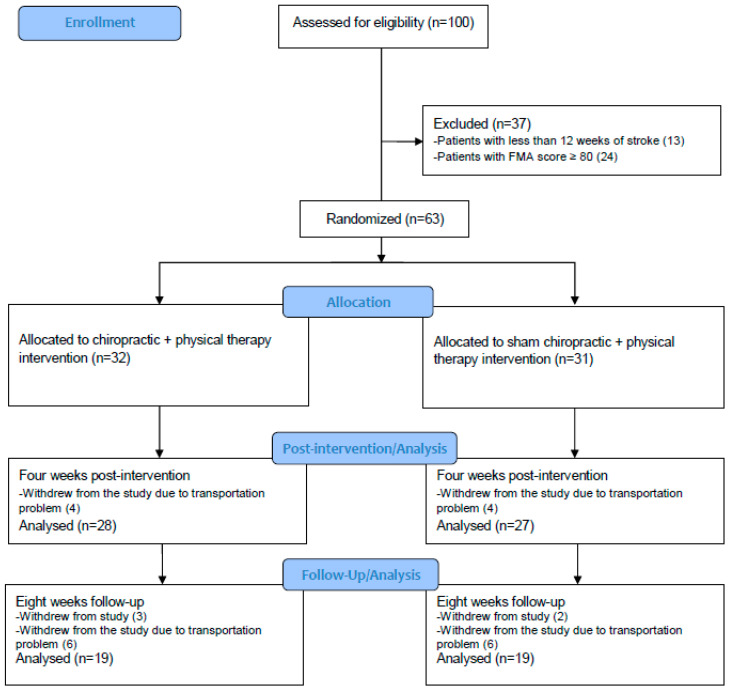
CONSORT study flow diagram. Abbreviations: Fugl-Meyer Assessment (FMA).

Out of 100 individuals with stroke who were screened for eligibility, 63 adults were eligible and were recruited between January and March 2019 (See study flow in Figure 1 and baseline demographic characteristics in Table 1). Fifty-five participants completed the four-week assessment and eight participants dropped out during the first four weeks of the study due to issues with caregiver availability or transportation limitations. There was a substantial number of dropouts between the four- and eight-week assessments that resulted in 38 participants completing the eight-week assessment. The loss-to-follow-up was due to the inability of some participants to stay away from their home longer than the duration of the active intervention, as they had travelled from surrounding regions with their caregivers so they could be involved in the study. There were no adverse events or reports of harm noted during the study. 

### 3.1. Within and between-Group Comparisons

Table 2 shows a significant two-way interaction between outcome and time, which means that the change of log-concentration across the three time points was different for the different blood biomarkers. For example, for IGF-II, the log concentration decreased across the time points, whereas, for GDNF, the log concentration followed an entirely different change across the time points. However, no significant interaction of Group at any level was present. The main effect of Group is also not significant. This suggests that there were no differences between the two groups in any of the blood biomarkers, at either the four- or eight-week time-points. The estimated marginal means for chiropractic plus physical therapy and sham chiropractic plus physical therapy at the three time points is given in Table 3.

### 3.2. Between-Group Differences

Table 4 summarizes the mean between group difference scores in log-concentration units. None of these mean differences were statistically significant. 

### 3.3. Within-Group Estimates

**Figure 2:**
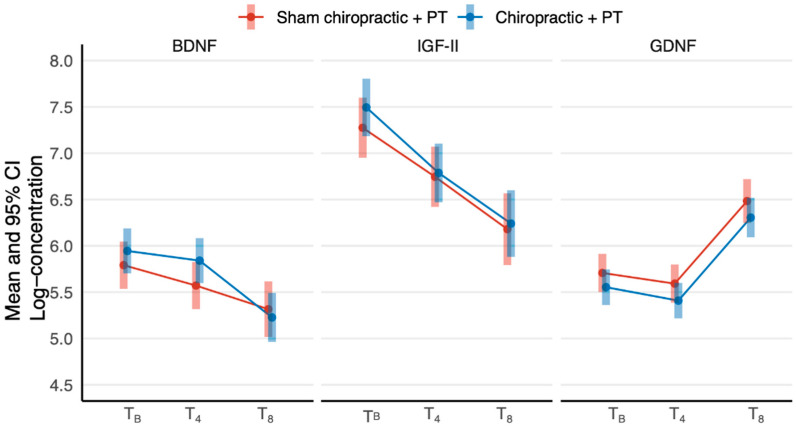
Estimated marginal means for chiro + PT and sham + PT at the three time points: baseline (TB), at four weeks (T4) and at eight weeks (T8). CI = confidence interval.

Within-group means along with their confidence intervals at the three time points are plotted in Figure 2. Mean log-concentration of BDNF and IGF-II significantly decreased over time at four and eight weeks in both chiro + PT and sham + PT groups. The log-concentration of GDNF did not vary from baseline to the four-week time point but significantly increased at the eight-week time point for both groups.

## 4. Discussion

These secondary RCT outcomes represent the first multi-session study to evaluate the effects of chiropractic adjustments on serum BDNF, IGF-II and GDNF in people with stroke. In both groups there was a significant decrease in the serum levels of BDNF and IGF-II over time, with a significant increase in serum GNDF levels at the eight-weeks follow-up. No significant between-group differences were found. This suggests that the addition of chiropractic care to a physical therapy program did not significantly alter the impact of physical therapy alone on these biomarkers.

The significant decrease in BDNF levels observed in the present study supports the findings from previous studies that showed decreased serum BDNF levels after single or multi-session aerobic exercise training in people with chronic stroke [88,89,90,91,92]. A significant decrease in serum BDNF was noted in people with chronic stroke who underwent a 36-session moderate-intensity (60% VO2 peak) continuous training program on a bicycle ergometer. Mild-intensity treadmill training [89,90], moderate-intensity continuous treadmill training [91], high-intensity treadmill training [89,90,92] and total-body ergometers [89] were also reported to decrease serum BDNF levels in people with chronic stroke. A significant reduction was also noted when serum BDNF levels were compared between week one and week three of acute inpatient stroke rehabilitation [93]. 

In contrast, other studies have found increased serum BDNF levels in individuals with stroke following rehabilitative programs. Eight weeks of aerobic exercise training after physical therapy in people with sub-acute and chronic stroke resulted in increased serum BDNF levels compared to physical therapy alone, which led to no significant change in serum BDNF levels [42]. One study in 2018 reported an increase in serum BDNF levels in people with chronic stroke after a single-session walking task [88]. Another recent study found that augmented reality-based rehabilitation consisting of motor rehabilitation using motion sensors and augmented reality significantly increased serum BDNF levels and improved motor function in people with sub-acute stroke [94]. An increase in serum BDNF levels has also been reported immediately after a single session of exercise in people with chronic stroke [88]. 

Brain-derived neurotrophic factor is an important regulator of neural regeneration and recovery [15,95,96,97]. An activity-driven increase in peri-infarct BDNF has been shown to promote motor recovery after stroke [98]. While the evidence regarding increases or decreases in BDNF levels with treatment is contradictory and somewhat confusing [99], there are several possible explanations for this predicament. Previous studies have suggested that increases in BDNF levels occur only after moderate-intensity physical exercise. Morais et al. (2018) reported a pre-post increase in serum BDNF levels in people with chronic stroke after a single session moderate-intensity walking task [88]. King et al. (2019) found no change in pre-post serum BDNF levels after a single session of incremental maximal aerobic exercise in people with chronic stroke [92]. The authors hypothesized that the participants’ reduced physical capacity might have prevented them from achieving the optimal intensity to induce a change in BDNF levels. Notably, increased problem-solving and reasoning ability (i.e., fluid intelligence) predicted larger increases in BDNF levels after exercise [92]. As similar brain areas are thought to be necessary for both fluid intelligence and cognition [100], impairment in cognition may influence the change in BDNF levels. However, a direct relationship between fluid intelligence and exercise dependent BDNF levels is yet to be explored. Inadequate exercise duration was speculated as another reason for the lack of change in BDNF levels, as the duration of the incremental maximal exercise was shorter than other protocols that reported an increase in BDNF [92]. A significant change with a large effect size was found when the average volume of hours spent exercising was over 20 ± 20 h in a three-to-eight-week program [41]. Whereas, those who exercised less, but over more weeks (12.9 ± 3.9 h over a length of four-to-24 weeks), did not exhibit any significant difference [41]. Studies on animals and healthy individuals have also shown that exercise-induced increases in BDNF levels require sufficient intensity [101] and duration [102] of exercise to significantly change. Therefore, exercise intensity, exercise duration and presence of cognitive impairment may be essential factors for exercise-induced changes in serum BDNF levels. In the present study, the intensity and duration of the interventions may have been insufficient to induce an increase in the serum BDNF levels. 

Another factor responsible for the decrease in BDNF level estimates may be the timing of BDNF measurement. An RCT on people with progressive multiple sclerosis reported a significant increase in serum BDNF levels after 30 min of bicycling, which decreased below baseline levels when measured 30 min post-exercise [103]. A systematic review also revealed that the increase in BDNF levels after acute aerobic exercise and/or training were not long-lasting in healthy individuals as well as those with chronic disease or disability [40]. A transient increase in peripheral BDNF was reported in 69% of the studies of healthy individuals and 86% of the studies of people with chronic disease or disability [40]. The lack of a long-lasting effect was speculated to be due to absorption of BDNF by central tissues (via the BBB) and use/clearance in the peripheral tissues, effectively normalizing BDNF levels post-exercise [40]. Therefore, the timing of BDNF measurement may explain the decrease in serum BDNF found in our study, as the blood samples were collected 30 min to 24 h after the completion of a four-week intervention program. 

Lastly, the observed decrease in BDNF levels could also be attributed to a mutation in the BDNF gene, referred to as Val66Met polymorphism. The BDNF val66met polymorphism impairs the beneficial effects induced by physical exercise [104] and results in 18% to 30% less activity-dependent release of BDNF [105]. In people with stroke, presence of the BDNF val66met polymorphism is associated with decreased brain activation in the primary sensorimotor cortex contralateral to the movement [106] as well as slower or reduced behavioral recovery from stroke [107]. In the present study, as the genotype of included participants was not evaluated, it is not known if the decrease in BDNF estimates was related to the BDNF val66met polymorphism. However, it must be noted that val66met is a common polymorphism present in humans and is more frequent in Asian populations compared to Caucasian ones [108]. Future studies may consider including genetic testing for val66met polymorphism to determine its influence on the findings. 

In the present study, a statistically significant decrease in IGF-II levels was noted in both groups across time. To date, there are no studies that have evaluated the effects of a single session of exercise or chiropractic care on serum IGF-II levels in people with stroke. Studies on healthy individuals are limited and have reported inconsistent findings. An RCT including 34 elderly Korean women found that 10 weeks of combined resistance and aerobic exercise increased IGF-II levels in the intervention group as compared to the control group [38], however, this study was limited as the results were inferred from the interaction effect (time × group) on the levels of IGF-II (F = 8.592, *p* = 0.006), rather than the main effect of groups. In addition, the lack of reporting on the blinding and randomization processes and the choice of statistical analysis (repeated analysis of variance for a between-subject design) further suggest that these results should be interpreted with caution. Studies evaluating the effect of a single exercise session on serum IGF-II have either reported no change [109] or an increase in IGF-II levels [110,111]. A crossover RCT including seven healthy men reported no change in levels of total IGF-II when evaluated immediately after a single, brief, high-intensity exercise, or after a delay of 10-, 20- or 30-min post-exercise [109]. In contrast, a crossover RCT [111] reported an increase in serum IGF-II in 10 healthy men when evaluated immediately after a single session of high-intensity exercise on a cycle ergometer. However, IGF-II returned to baseline levels when evaluated after 10-, 20-, 30-, 40- and 50-min post-exercise [111]. The authors speculated that the change in serum IGF-II levels may have been dependent on the intensity of exercise as the increase in serum IGF-II estimates with low-intensity exercise did not reach statistical significance [111]. A significant increase was also noted with a single session of moderate-intensity endurance exercise when six untrained healthy individuals were evaluated 10 min post-exercise [112]. However, based on the literature on IGF-I levels in people with stroke, a related blood marker, the decrease in IGF-II observed in our study may be due to absorption of IGF-II across the BBB [112]. More studies are needed to further understand changes observed in IGF-II in people with stroke. 

Lastly, significantly increased GDNF levels were noted at the eight-week follow-up in both groups in the present study. The function and expression of GDNF could be regulated by physical activity. Studies in rodents have shown that short-term and long-term exercise increases the expression of GDNF in different CNS structures [35,113]. In one of these studies, GDNF increased significantly in the striatum corresponding to the overused limb subjected to forced limb use by applying a cast on the other limb [35]. Another study showed that both passive and active exercise increased GDNF in the spinal cord of young rats [113]. Whereas in stroke, due to the occlusion of the middle cerebral artery in rats, GDNF receptors were upregulated in the cerebral cortex and striatum, which are two structures affected by lack of blood supply [34,114].

### Limitations, Implications and Future Research 

A limitation of the present study was that blood samples were not evaluated immediately after each intervention, yet instead were collected at four-week intervals, thus not revealing any potential acute post-adjustment or post-exercise changes. As activity-induced changes in biomarkers may be transient, particularly for BDNF [40], evaluating the time course of change in biomarkers can be explored in further research. The present study did not track patients’ heart rate, VO2max or other measures of exercise intensity, which could have affected the magnitude or directionality of change in biomarkers. This study is also limited by the lack of information on participants’ genotypes, for example, the presence or absence of the BDNF val66met polymorphism. As this study included chronic stroke patients of a mean age of over 50, it is possible that physical and/or cognitive impairments limited participants’ exercise intensity and subsequent biomarker changes. The present study lacked an a-priori sample size calculation for the studied secondary outcomes, as the sample was designed to sufficiently power the primary objectives in the main RCT.

The secondary outcomes in the present study explored the potential of combining chiropractic adjustments with physical therapy to change serum BDNF, GDNF and IGF-II levels in people with chronic stroke. In our previously published RCT, which included the same 63 patients with stroke, statistically significant improvements in motor function were seen in both chiro + PT and sham + PT groups [70]. Importantly, the chiro + PT group had greater motor function improvements than sham + PT [70]. However, the current study did not show any differences between chiro + PT compared to PT alone on serum BDNF, GDNF and IGF-II levels. The current study findings therefore suggest that the previously demonstrated greater improvements in motor function in the chiro + PT group are not the result of changes in BDNF, GDNF and IFG-II. However, we cannot rule out that the overall change seen in blood biomarkers in both groups could relate to the motor improvements seen in both groups in the main RCT. Further research is needed to elucidate the factors that influence changes in BDNF, GDNF and IGF-II levels in people with stroke receiving physical therapy and chiropractic adjustments.

## 5. Conclusions

The present secondary RCT outcomes demonstrated significant changes in BDNF, GDNF and IGF-II levels in people with chronic stroke who received a chiropractic + physical therapy intervention and in those who received a sham chiropractic + physical therapy intervention. However, including chiropractic spinal adjustments in the intervention did not cause significant differences in these changes. These findings suggest that motor function improvements following chiropractic spinal adjustment in individuals with stroke are not explained by changes in BDNF, GDNF and IGF-II. The changes in BDNF, GDNF and IGF-II in both groups may be related to several variables including the intensity and duration of the intervention, timing of measurement, genetic polymorphism and cognitive impairment, which may be clarified in future studies.

## Figures and Tables

**Table 1:** Clinical characteristics of participants in each group.

Variables	Chiro + PT	Sham + PT
Gender		
Male (*n*)	18	16
Female (*n*)	10	11
Age, years (mean ± SD)	53.3 ± 14.0	58.5 ± 11.3
Side of body affected by stroke		
Left (*n*)	14	12
Right (*n*)	14	15
Time since stroke, months (mean ± SD)	30.0 ± 36.6	27.3 ± 31.5
12–24 weeks, *n*	5	4
>24 weeks, *n*	23	23
Type of stroke		
Ischemic (*n*)	24	25
Hemorrhagic (*n*)	4	2

*n*: number of participants, SD: standard deviation.

**Table 2:** Multivariate analysis of variance table with tests for variance explained by the categorical independent variables and their interactions.

Independent Variable	DF_num_	DF_den_	F-Value	*p*-Value
Outcome	3	276	1836.18	<0.001
Time	2	276	3.66	0.027
Group	1	40	0.76	0.39
Outcome × Time	4	276	16.56	<0.001
Outcome × Group	2	276	1.52	0.22
Time × Group	2	276	1.18	0.31
Outcome × Time × Group	4	276	0.49	0.75

DF_num_ = numerator degrees of freedom, DF_den_ = denominator degrees of freedom.

**Table 3:** Estimated marginal means for chiropractic plus physical therapy and sham chiropractic plus physical therapy at the three time points.

Outcome	Group	Time	Mean ± SE	95% CI
BDNF	Sham chiropractic + PT	Baseline	5.8 ± 0.1	5.5, 6.0
	Chiropractic + PT	Baseline	5.9 ± 0.1	5.7, 6.2
	Sham chiropractic + PT	At 4 weeks	5.6 ± 0.1	5.3, 5.8
	Chiropractic + PT	At 4 weeks	5.8 ± 0.1	5.6, 6.1
	Sham chiropractic + PT	At 8 weeks	5.3 ± 0.2	5.0, 5.6
	Chiropractic + PT	At 8 weeks	5.2 ± 0.1	5.0, 5.5
IGF-II	Sham chiropractic + PT	Baseline	7.3 ± 0.2	7.0, 7.6
	Chiropractic + PT	Baseline	7.5 ± 0.2	7.2, 7.8
	Sham chiropractic + PT	At 4 weeks	6.7 ± 0.2	6.4, 7.1
	Chiropractic + PT	At 4 weeks	6.8 ± 0.2	6.5, 7.1
	Sham chiropractic + PT	At 8 weeks	6.2 ± 0.2	5.8, 6.6
	Chiropractic + PT	At 8 weeks	6.2 ± 0.2	5.9, 6.6
GDNF	Sham chiropractic + PT	Baseline	5.7 ± 0.1	5.5, 5.9
	Chiropractic + PT	Baseline	5.6 ± 0.1	5.4, 5.7
	Sham chiropractic + PT	At 4 weeks	5.6 ± 0.1	5.4, 5.8
	Chiropractic + PT	At 4 weeks	5.4 ± 0.1	5.2, 5.6
	Sham chiropractic + PT	At 8 weeks	6.5 ± 0.1	6.2, 6.7
	Chiropractic + PT	At 8 weeks	6.3 ± 0.1	6.1, 6.5

SE = standard error and CI stands for confidence interval.

**Table 4:** Mean differences between chiro + PT and sham + PT in log-concentration of bloodwork biomarkers.

Outcome	Time	Mean Difference ± SE	95% CI	H_0_: Mean Difference = 0, t [df], *p*-Value
BDNF	At 4 weeks	0.12 ± 0.21	−0.31, 0.54	0.54 [276], 0.59
	At 8 weeks	−0.24 ± 0.23	−0.70, 0.22	−1.03 [276], 0.30
IGF-II	At 4 weeks	−0.18 ± 0.29	−0.75, 0.40	−0.61 [276], 0.54
	At 8 weeks	−0.16 ± 0.32	−0.79, 0.47	−0.50 [276], 0.62
GDNF	At 4 weeks	−0.03 ± 0.16	−0.35, 0.29	−0.18 [276], 0.86
	At 8 weeks	−0.03 ± 0.18	−0.38, 0.33	−0.14 [276], 0.89

SE = standard error, CI = confidence interval, df = degrees of freedom, H_0_ = the null hypothesis. Mean difference is defined as [chiro + PT at each assessment—chiro + PT at baseline]—[sham + PT at each assessment -sham + PT as baseline].

## Data Availability

Reasonable requests for data can be made to the corresponding author but ethics committee approval will needed to be granted prior to sharing any data.

## Supplementary Materials

The following supporting information can be downloaded at: https://www.mdpi.com/article/10.3390/jcm11247493/s1, File S1: Stats Reort:The effects of four weeks of chiropractic spinal adjustments on blood biomarkers in adults with chronic stroke: A randomized controlled trial. File S2: CONSORT 2010 checklist of information to include when reporting a randomised trial.
