# Supplementary material for: The Effects of Four Weeks of Chiropractic Spinal Adjustments on Blood Biomarkers in Adults with Chronic Stroke: Secondary Outcomes of a Randomized Controlled Trial
Source: J Clin Med. 2022 Dec 17;11(24):7493. doi: 10.3390/jcm11247493 (PMC9786914; doi:10.3390/jcm11247493)

Stats Reort:The effects of four weeks of chiropractic spinal adjustments on blood biomarkers in adults with chronic stroke: A randomized controlled trial

Usman Rashid

Contents

1 Data Visualisations **3**
1.1 Data Normality Check and Log Transformation . . . 3
1.2 Box and Scatter Plots . . . 5

2 Statistical Model **7**
2.1 Multivariate Analysis of Variance . . . 7
2.2 Diagnostics for the Model . . . 7

3 Results **8**
3.1 MANOVA Table . . . 8
3.2 Estimated Means and their Contrasts . . . 8
3.3 Estimated Means Plot . . . 9

1 Data Visualisations

1.1 Data Normality Check and Log Transformation

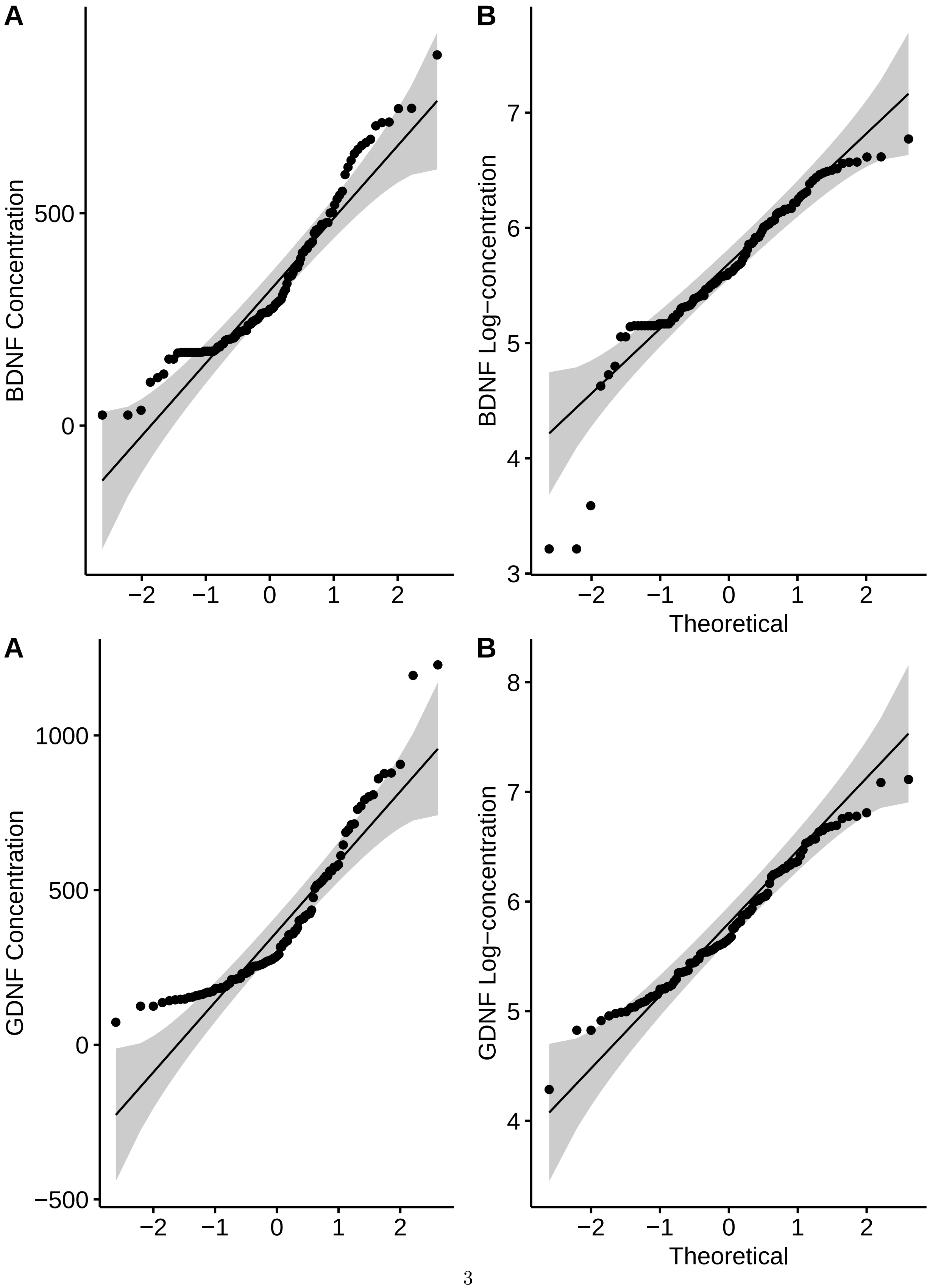

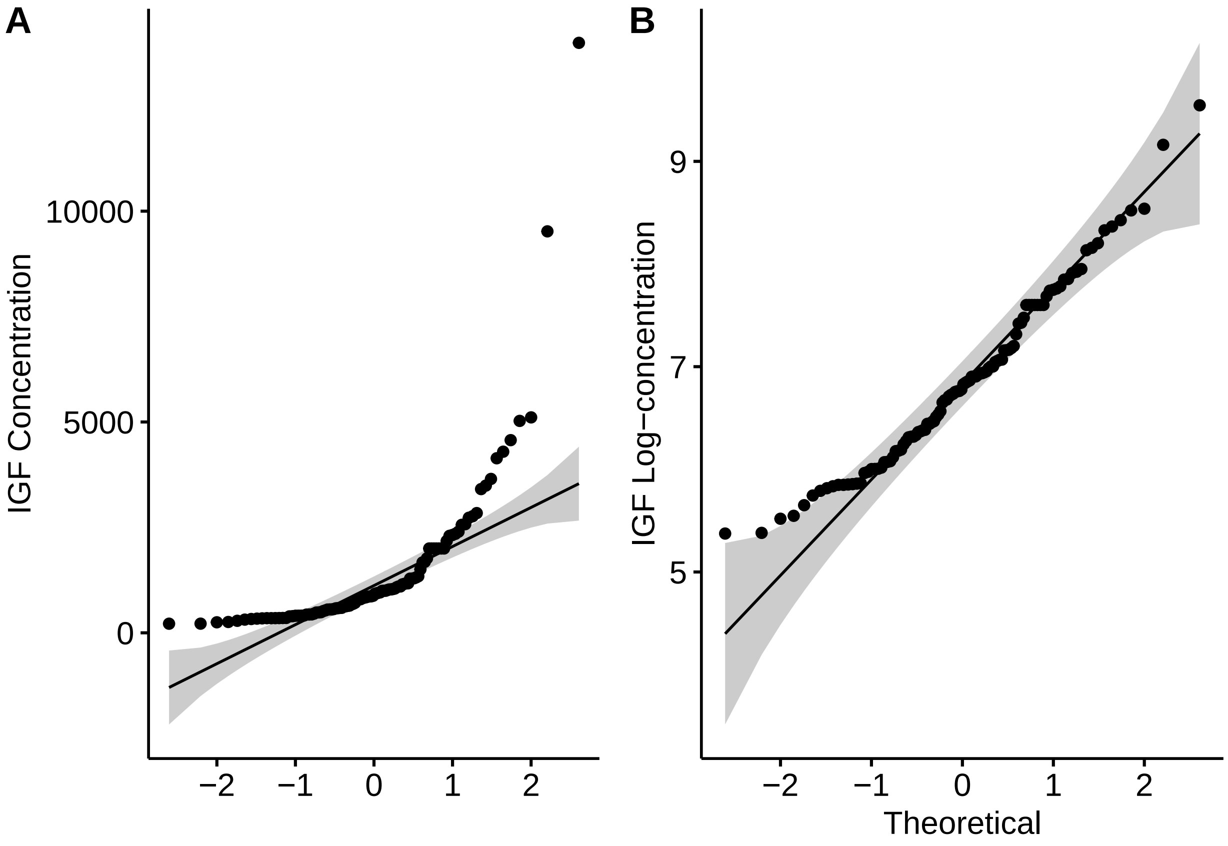

A
B
IGF Concentration
10000
5000
0
−2
−1
0
1
2
IGF Log−concentration
9
7
5
−2
−1
0
1
2
Theoretical

1.2 Box and Scatter Plots

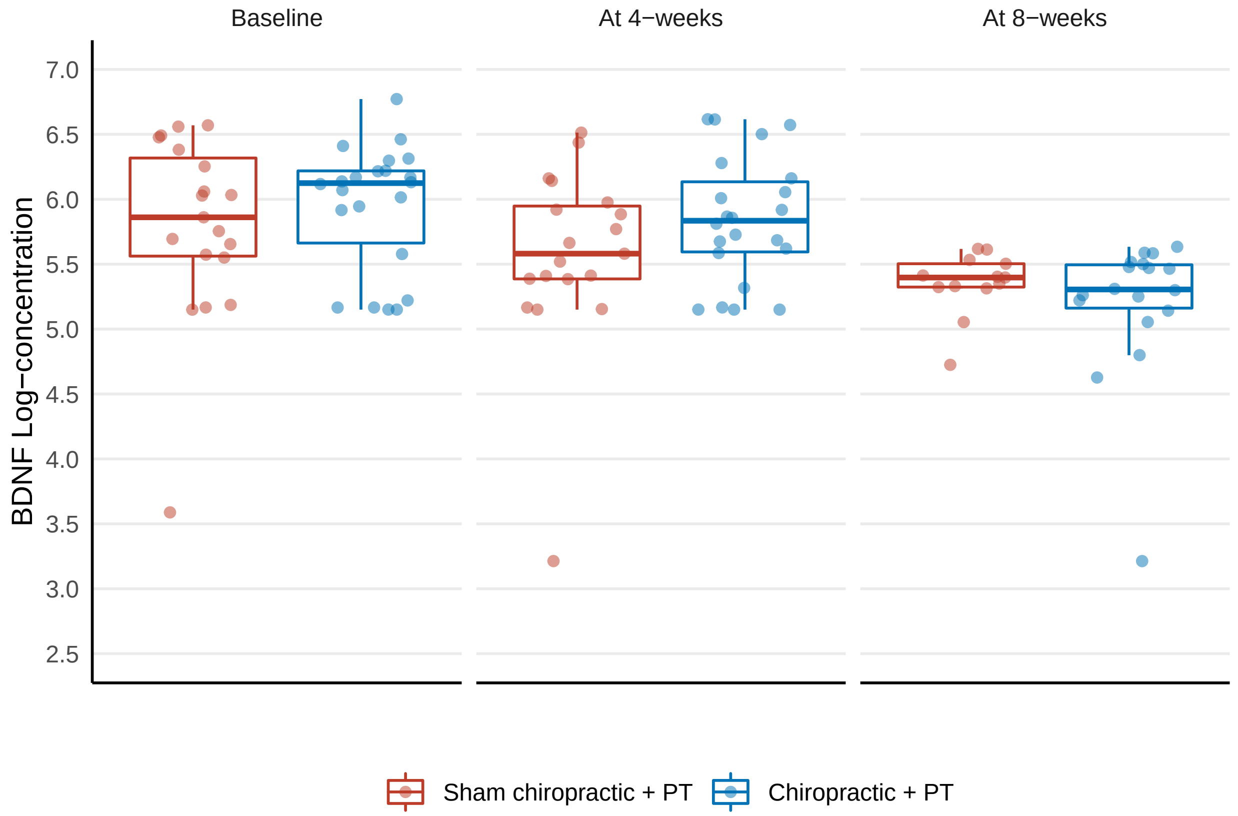

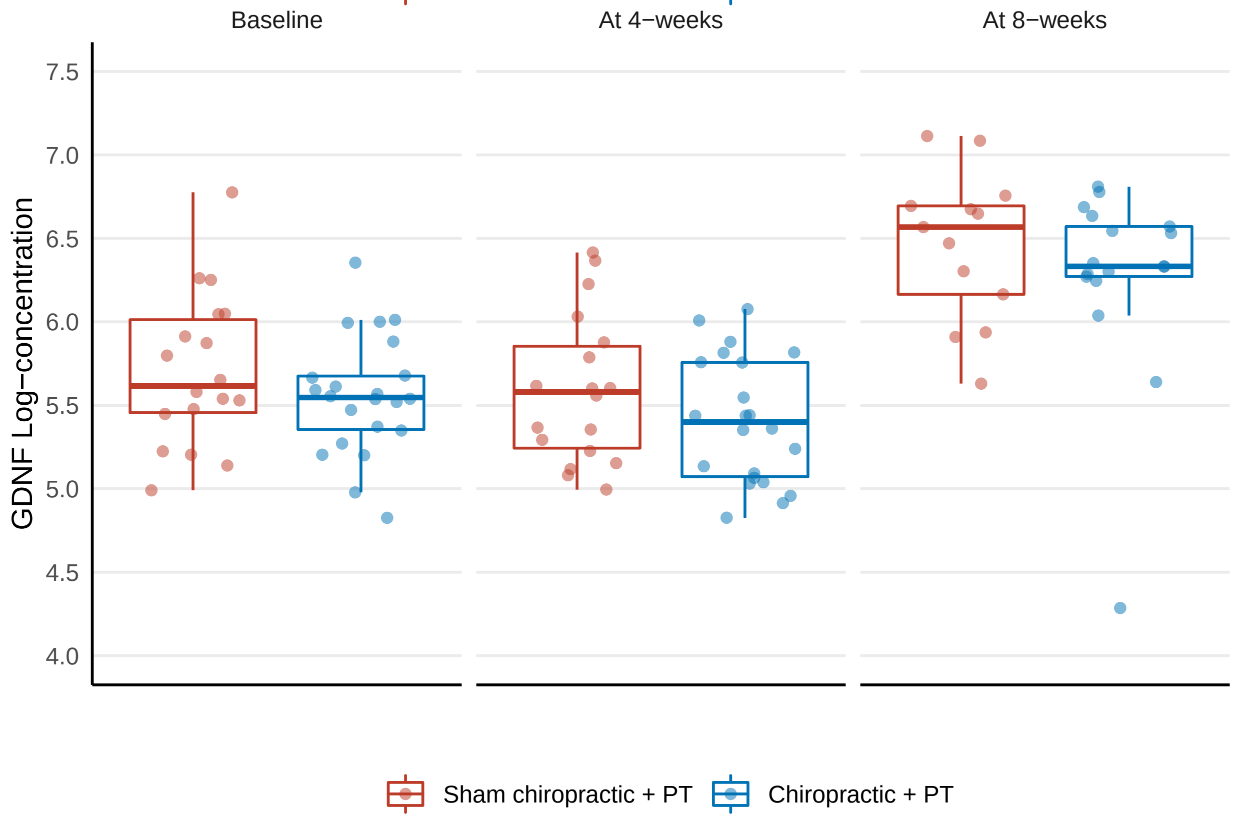

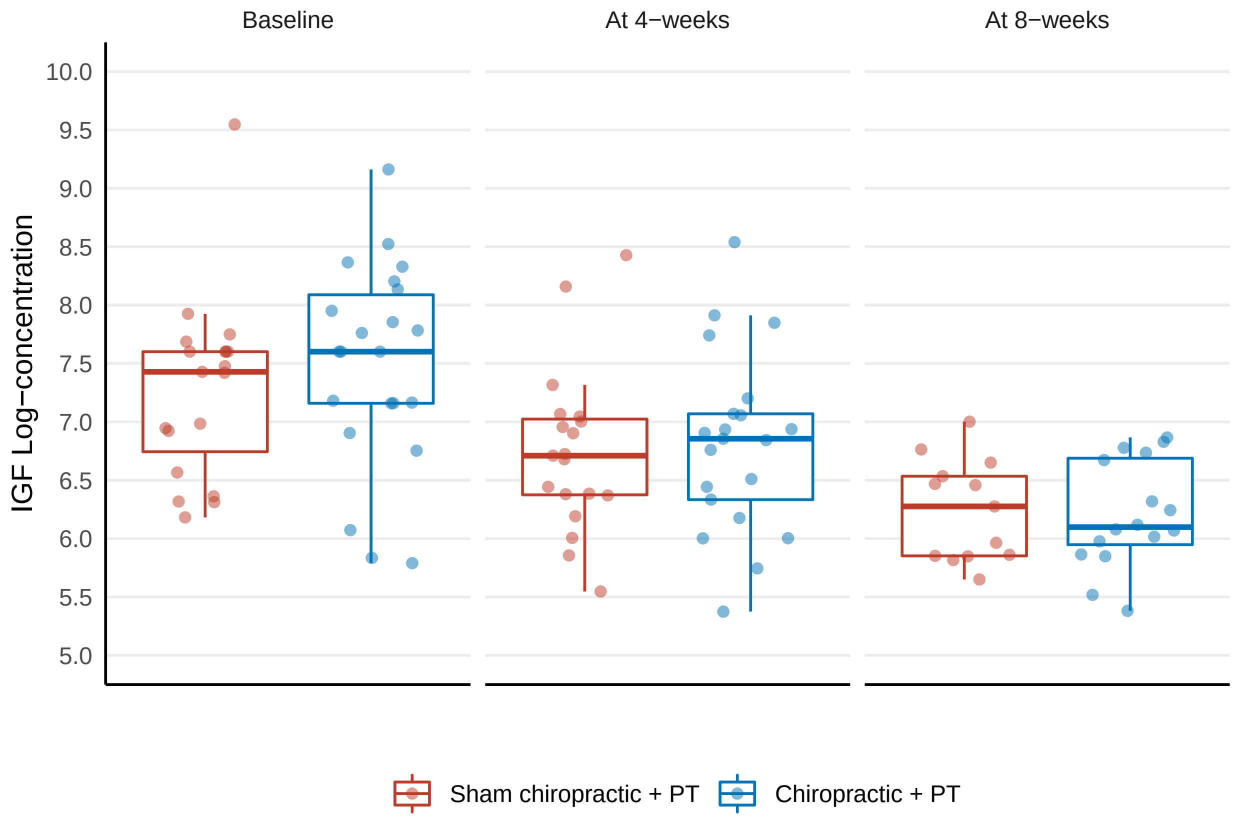
Baseline
At 4−weeks
At 8−weeks
IGF Log−concentration
10.0
9.5
9.0
8.5
8.0
7.5
7.0
6.5
6.0
5.5
5.0
Sham chiropractic + PT
Chiropractic + PT

2 Statistical Model

2.1 Multivariate Analysis of Variance

```
lmerModel.multi  <- lme(log(Concentration) ~ 0+Outcome*Time*Group,
                        random = ~ 0+Outcome|PartId,
                        Datasource.all,
                        na.action = na.omit,
                        weights = varIdent(form = ~ 1|Outcome),
                        control = lmeControl(maxIter = 1e3,
                                             msMaxIter = 1e3,
                                             niterEM = 1e3,
                                             msMaxEval = 1e3))
```

2.2 Diagnostics for the Model

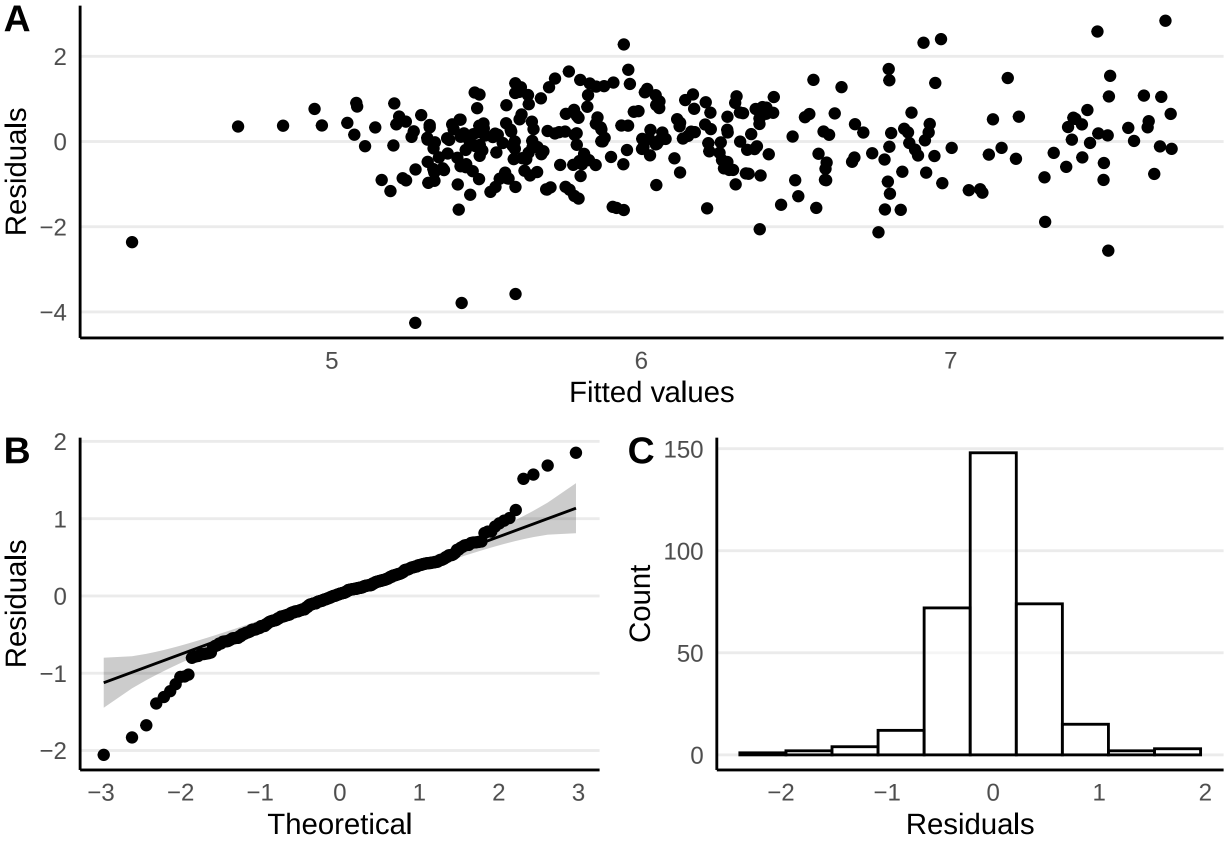

3 Results

3.1 MANOVA Table

	numDF	denDF	F-value	p-value
Outcome	3	276	1836.1791798	0.0000000
Time	2	276	3.6547016	0.0271292
Group	1	40	0.7579630	0.3891619
Outcome:Time	4	276	16.5596953	0.0000000
Outcome:Group	2	276	1.5225641	0.2199784
Time:Group	2	276	1.1820079	0.3082098
Outcome:Time:Group	4	276	0.4873873	0.7450067

3.2 Estimated Means and their Contrasts

Mean difference (MD) is defined as [Chiropractic + PT – (Chiropractic + PT)Baseline] – [PT – (PT)Baseline].

Outcome	Group	Time	Mean	SE	95% CI lower	95% CI upper
BDNF	Sham chiropractic + PT	Baseline	5.8	0.1	5.5	6.0
BDNF	Chiropractic + PT	Baseline	5.9	0.1	5.7	6.2
BDNF	Sham chiropractic + PT	At 4-weeks	5.6	0.1	5.3	5.8
BDNF	Chiropractic + PT	At 4-weeks	5.8	0.1	5.6	6.1
BDNF	Sham chiropractic + PT	At 8-weeks	5.3	0.2	5.0	5.6
BDNF	Chiropractic + PT	At 8-weeks	5.2	0.1	5.0	5.5
GDNF	Sham chiropractic + PT	Baseline	5.7	0.1	5.5	5.9
GDNF	Chiropractic + PT	Baseline	5.6	0.1	5.4	5.7
GDNF	Sham chiropractic + PT	At 4-weeks	5.6	0.1	5.4	5.8
GDNF	Chiropractic + PT	At 4-weeks	5.4	0.1	5.2	5.6
GDNF	Sham chiropractic + PT	At 8-weeks	6.5	0.1	6.2	6.7
GDNF	Chiropractic + PT	At 8-weeks	6.3	0.1	6.1	6.5
IGF	Sham chiropractic + PT	Baseline	7.3	0.2	7.0	7.6
IGF	Chiropractic + PT	Baseline	7.5	0.2	7.2	7.8
IGF	Sham chiropractic + PT	At 4-weeks	6.7	0.2	6.4	7.1
IGF	Chiropractic + PT	At 4-weeks	6.8	0.2	6.5	7.1
IGF	Sham chiropractic + PT	At 8-weeks	6.2	0.2	5.8	6.6
IGF	Chiropractic + PT	At 8-weeks	6.2	0.2	5.9	6.6

Outcome	Contrast	Mean	SE	95% CI lower	95% CI upper	DF	t-Value	P-value
BDNF	MD At 4-weeks	0.12	0.21	-0.31	0.54	276	0.54	0.59
BDNF	MD At 8-weeks	-0.24	0.23	-0.70	0.22	276	-1.03	0.30
GDNF	MD At 4-weeks	-0.03	0.16	-0.35	0.29	276	-0.18	0.86
GDNF	MD At 8-weeks	-0.03	0.18	-0.38	0.33	276	-0.14	0.89
IGF	MD At 4-weeks	-0.18	0.29	-0.75	0.40	276	-0.61	0.54
IGF	MD At 8-weeks	-0.16	0.32	-0.79	0.47	276	-0.50	0.62

3.3 Estimated Means Plot

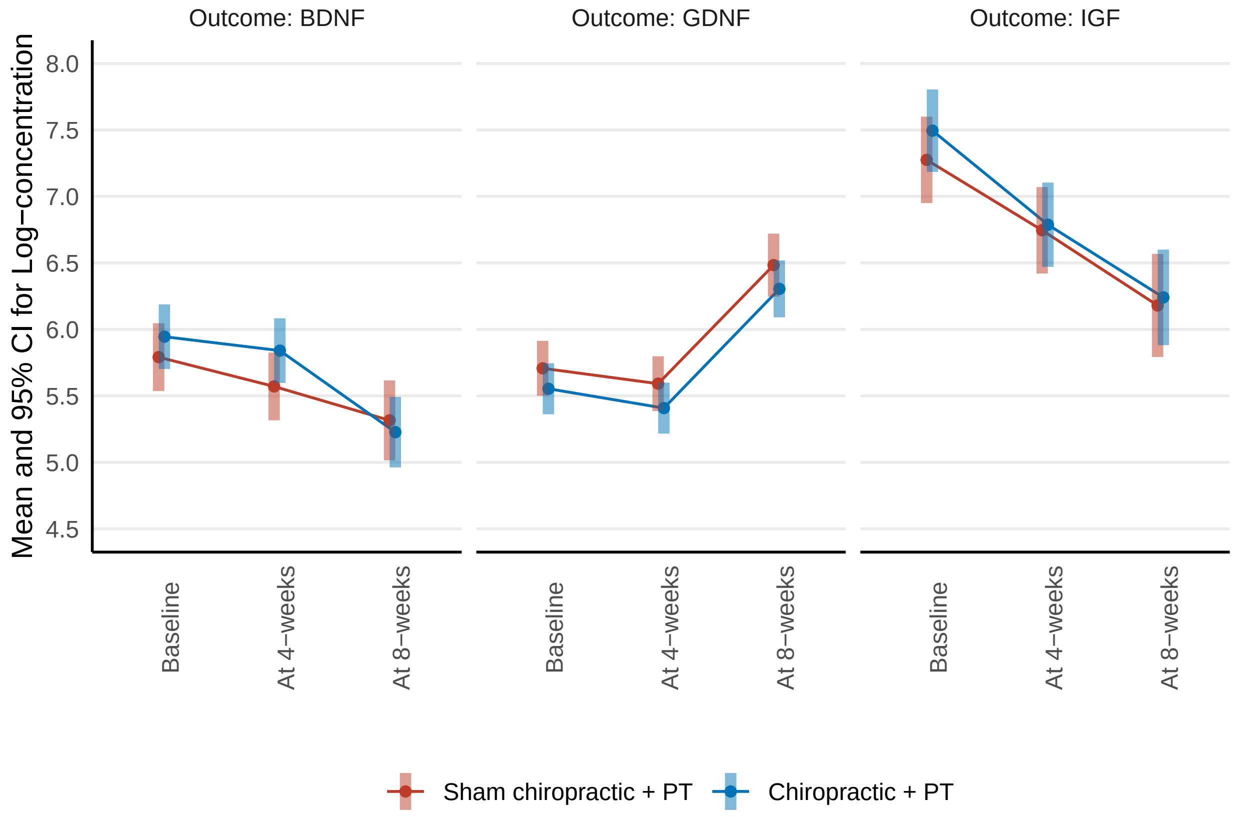

Supplement: Supplementary file 1 [file jcm-11-07493-s001.zip › jcm-2099178-supplementary.pdf]
